# On the censored cost-effectiveness analysis using copula information

**DOI:** 10.1186/s12874-017-0305-9

**Published:** 2017-02-15

**Authors:** Charles Fontaine, Jean-Pierre Daurès, Paul Landais

**Affiliations:** UPRES EA2415-Institut Universitaire de Recherche Clinique, Université de Montpellier, 641, Av. du doyen G.-Giraud, Montpellier, France

**Keywords:** Cost-effectiveness analysis, Censored data, Copulas, Parametric models, Subgroups analysis

## Abstract

**Background:**

Information and theory beyond copula concepts are essential to understand the dependence relationship between several marginal covariates distributions. In a therapeutic trial data scheme, most of the time, censoring occurs. That could lead to a biased interpretation of the dependence relationship between marginal distributions. Furthermore, it could result in a biased inference of the joint probability distribution function. A particular case is the cost-effectiveness analysis (CEA), which has shown its utility in many medico-economic studies and where censoring often occurs.

**Methods:**

This paper discusses a copula-based modeling of the joint density and an estimation method of the costs, and quality adjusted life years (QALY) in a cost-effectiveness analysis in case of censoring. This method is not based on any linearity assumption on the inferred variables, but on a punctual estimation obtained from the marginal distributions together with their dependence link.

**Results:**

Our results show that the proposed methodology keeps only the bias resulting statistical inference and don’t have anymore a bias based on a unverified linearity assumption. An acupuncture study for chronic headache in primary care was used to show the applicability of the method and the obtained ICER keeps in the confidence interval of the standard regression methodology.

**Conclusion:**

For the cost-effectiveness literature, such a technique without any linearity assumption is a progress since it does not need the specification of a global linear regression model. Hence, the estimation of the a marginal distributions for each therapeutic arm, the concordance measures between these populations and the right copulas families is now sufficient to process to the whole CEA.

## Background

Due to the variety of treatments for a specific health problem and in conjunction with their increasing costs, cost-effectiveness studies of new therapies is challenging. These studies could achieve to a statistical analysis since that the common practice in laboratories is to collect individual patient cost data in randomized studies. Furthermore, it is now possible to compute the incremental net benefit from the use of a new therapy in comparison with the common-in-use therapy.

In the last decades, the cost-effectiveness analysis (CEA) of new treatments became an actual subject of work for statisticians. It is used in two particular designs: decision modeling-based CEA and trial-based CEA. The major difference between both approaches is that in the case of trial-based CEA, data are gathered at the patients level in particular studies and it may lead to overlearning from the study, which may lead to mistakes in interpretation when the results are inferred for populations. In contrast, decision modeling-based CEA are based on easily generalizable data.

The cost-effectiveness analysis is used to measure the incremental cost-effectiveness ratio (ICER) and the incremental net benefit (INB). The ICER is defined as the ratio:

$$\begin{array}{@{}rcl@{}} ICER&=& \frac{\mathbb{E}(C_{1})-\mathbb{E}(C_{0})}{\mathbb{E}(T_{1})-\mathbb{E}(T_{0})} \end{array} $$

where *C*_1_ is the cost of the tested therapeutic, *C*_0_ is the cost of the control group which is usually measured in term of a given monetary unit, *T*_1_ is the effectiveness of the tested therapeutic which is usually measured in term of survival life years, *T*_0_ is the effectiveness of the control group and, $\mathbb {E}(\bullet)$ is the expectation function. Therefore, it is an indicator of the monetary cost of using a new therapy in terms of survival time. On the other hand, the INB is defined as the following difference:

$$\begin{array}{@{}rcl@{}} INB &=& \lambda (T_{1}-T_{0})-(C_{1}-C_{0}) \end{array} $$

where *λ* is the willingness-to-pay for a unit of effectiveness.

In the literature, many articles propose ways to estimate these quantities. At first, Willan and Lin [1] proposed an approach which is based on the sample mean. It was applied directly to survival time years. In case of censored data, they proposed to estimate the survival function for each arm using the product-limit estimator [2] and then to estimate the survival time by integrating the survival functions until a time *τ* (i.e. *μ*_*j*_ is the life expectancy until *τ*), the maximal time of follow-up. Concerning the estimation of costs in case of censoring, many estimators may be used. Zhao et al. [3] have shown the equivalence among them.

The quality adjusted life years (QALY) concept was introduced in 1977 by Weinstein and Stason [4], and is still actually one of the most important notions in the cost-effectiveness theoretical framework. In the paper of Willan et al. [5], the concept of quality of life adjusted to the survival time is defined as follows. Let *q*_*ji*_ be the quality adjusted survival for the period of interest for the patient *i* which follows the treatment *j* and let *q*_*jki*_ be the observed quality of life for the patient *i* receiving treatment *j*, *j*=0,1 during the interval of time *a*_*k*_. In fact, this is a representation of the standard survival times scaled down by the quality of life experienced by patients. One note that the duration of interest of a study (0,*τ*] is divided in *K* (arbitrary, relative to the data) sub-intervals [ *a*_*k*_,*a*_*k*+1_) where *k*=1,2,…,*K* and where 0=*a*_1_<*a*_2_<…<*a*_*K*+1_=*τ*. Thus, one can determine the value of *q*_*jki*_ in the following path: let a patient *i* be on treatment *j* with *B*_*ji*_ quality of life measured at times $\phantom {\dot {i}\!}t_{ji1},t_{ji2},\ldots,t_{{jim}_{{ji}}}$ with respective scores $\phantom {\dot {i}\!}Q_{ji1},Q_{ji2},\ldots,Q_{{jim}_{{ij}}}$. These scores are nothing more than the utility values. Therefore, $q_{{jki}}=\int _{a_{k}}^{a_{k+1}} Q(t)dt$ is a weighted sum of times spent in the different quality of life states where:

$${} Q(t) = \left \{ \begin{array}{ll} Q_{ji1} & \text{if} \,\,0\leq t < t_{ji1}; \\ Q_{jih}+ \frac{(Q_{ji,h+1}-Q_{jih})(t_{ji,h+1}-t_{jih})}{t_{ji,h+1}-t_{jih}} & \text{if}\,\, t_{jih}\leq t < t_{ji,h+1}; \\ Q_{{jim}_{ji}} & \text{if} \,\, t_{{jim}_{ji}}\leq t < X_{ji}; \\ 0 & \text{if} \,\, \geq X_{ji}, \end{array}\right. $$

and where  where *η* represents the censoring random variable. Furthermore, let  which indicates if a patient *i* on the treatment arm *j* is alive at time *a*_*k*_ and is not censored on [*a*_*k*_,*a*_*k*+1_) and let $Y_{jk}=\sum _{i=1}^{n_{j}}Y_{jki}$. If one notes $\bar {q}_{jk}=\sum _{i=1}^{n_{j}}(Y_{jki}q_{jki})/Y_{jk}$,then using a known expression of variance [5], one obtains the estimation of *μ*_*j*_, the expected value of effectiveness adjusted to QALY, with

$$\begin{array}{@{}rcl@{}} \hat{\mu}_{j} &=& \sum_{k=1}^{K} \hat{S}_{j}(a_{k})\bar{q}_{jk}. \end{array} $$

More recently, Willan et al. [6] proposed to realize the whole cost-effectiveness analysis using linear regression methods. Let *C*_*i*_ be the observed cost for patient *i*, then $\mathbb {E}(C_{ji})=\beta _{C_{i}}^{T}Z_{C_{ji}}, i=1,2,\ldots,n_{j}, Z_{C_{ji}}$ is a vector of covariates affecting costs and $\beta _{C_{j}}$ is a vector of unknown regression parameters. Then, using the inverse probability of censoring weighting (IPCW) methods, they proposed a way to estimate the second component of $\beta _{C_{j}}$ which is the mean difference in costs between randomization groups adjusted to other covariates, $\hat {\Delta }_{c}$, and its associate variance. The same methodology is done there to estimate the mean difference in mean survival between randomization groups adjusted for the other covariates, $\hat {\Delta }_{e}$. Thus, they proposed to estimate the ICER adjusted for the quality of life by $\hat {\Delta }_{c}/\hat {\Delta }_{e}$ and used this time again the Fieller’s theorem to find a 100(1−*α*) confidence interval. For the adjusted INB, they proposed to estimate it by $\hat {b}_{\lambda }=\lambda \hat {\Delta }_{e}-\hat {\Delta }_{c}$ with variance $\hat {\sigma }_{\lambda }^{2}=\lambda ^{2}\hat {\sigma }^{2}_{\Delta _{e}}+\hat {\sigma }^{2}_{\Delta _{c}}-2\lambda \hat {\sigma }_{\Delta _{c}\Delta _{e}}$. Thus, if the INB is positive, the therapy is cost-effective and if it is negative, there is no cost-effectiveness. One remarks that it is possible to determine the cost-effectiveness under a significance level *α* using a statistic of test: if $\hat {b}_{\lambda }/\hat {\sigma }_{\lambda }$ is greater than the test level *z*_1−*α*_, at a level *α*, the therapy is cost-effective compared to the standard.

From that linear regression approach, many variants exist either parametric or semi-parametric [7]. The main problem of the estimator for cost-effectiveness analysis based on the linear regression is that even if the inverse-probability censored weighting estimator is consistent, it is not efficient because if an individual is censored before or at time *a*_*K*+1_, the patient does not contribute to the sum that constitutes this estimator and the practitioner may lose some statistical significant information. Furthermore, when there is no censoring, it is equivalent to the solve ordinary least squares equations; for which the linearity assumption may lead to serious bias in case of non-linearity. In the case of a semi-parametric estimator, the main problem is that the estimation of the conditional moments may be hard to find accurately or may lead to some bias in function of the used estimator. For these reasons, we introduce a new cost-effectiveness analysis methodology and a modeling of the joint density function between costs and QALY using parametric copulas. It is therefore based only on the dependence between covariates and the prior information on the variables distributions.

## Methods

### Copula function

To introduce the theory beyond the new proposed estimator for cost-effectiveness analysis, it is crucial to present the concept of modeling the dependence among two or more variables, namely the copula function concept. The idea of the copula started with Sklar [8] who formulated his famous theorem as follows: a d-dimensional multivariate distribution $H(x_{1},x_{2},\ldots,x_{d})=\mathbb {P}(X_{1}\leq x_{1}, X_{2}\leq $ *x*_2_,…,*X*_*d*_≤*x*_*d*_) from a random vector (*X*_1_,*X*_2_,…,*X*_*d*_)^*T*^ with marginal distributions $F_{i}(x)=\mathbb {P}(X_{i} \leq x)$ can be written as:

$$ H(x_{1},x_{2},\ldots,x_{d})=C(F_{1}(x_{1}),F_{2}(x_{2}),\ldots,F_{d}(x_{d})) $$

where *C* is the cumulative distribution functionof the copula. In fact, it is a cumulative distribution function from [0,1]^*d*^ to [0,1] with uniform margins over the unit interval [0,1] such that: $C(u_{1},u_{2},\ldots,u_{d})=\mathbb {P}(F_{1}(X_{1})\leq u_{1},F_{2}(X_{2})\leq u_{2},\ldots,F_{d}(X_{d})\leq u_{d})$. Therefore, the copula density can be written as follows:

$$ c(u_{1},u_{2},\ldots,u_{d})=\frac{\partial^{d} C(u_{1},u_{2},\ldots,u_{d})}{\partial u_{1},u_{2},\ldots,u_{d}}. $$

Thus, for a bivariate model, the joint density function of stochastic variables *X*_1_ and *X*_2_ is:

$$f(x_{1},x_{2})=c(F_{1}(x_{1}),F_{2}(x_{2}))f_{1}(x_{1})f_{2}(x_{2}). $$

Note that parametric and nonparametric copulas estimation exists. Also, one has to remark that given two marginal distributions, the copula that joins them is unique if and only if these margins are continuous. In the literature, few papers deal with copula and costs data. At first, Embrechts et al. [9] present an original article about the correlation measurement with those data. However, the copula concepts presented there are more about generalizations of the copula theory than about a modeling methodology. Secondly, Frees and Valdez [10] use copulas for costs data in the insurance area. Their work explains how to fit copulas and measure the mortality risks. Thirdly, there is Hougaard [11] who uses copula with costs data in a multivariate survival analysis context. Also, there is Zhao and Zhou who work with copula models using medical costs, but in a stochastic process context, which is not adapted to QALY and costs data when the information is limited.

In this paper, we assume the copula to be parametric, which means that the copula can be written in a particular way as a function of the chosen family, with one parameter who summarizes the dependence between the variables, and the marginal distributions to be continuous. For more about copulas, see Nelsen [12].

### Model

#### Determination of QALY in terms of time and quality of life

If the survival time adjusted for the quality of life is already measured, one should directly estimate the parameters of its distribution. However, most of the time, practitioners only have two variables: time and quality of life. As shown in the previous section, the classical adjustment method for time on quality of life is given by $T_{adj}(w)=\int _{0}^{T(w)} Q(v(t))dt$ where *Q*(*v*(*t*)) is the adjustment of quality of life scores on the interval of time of interest. Since that the function $H(t)=\int _{0}^{t} Q(v(y))dy$ is monotonically increasing, it is possible to rewrite the cumulative distribution function of *T*_*adj*_ as a composition of functions such as:

$$F_{adj}(y)=F \circ H^{-1}(y) $$

where $H^{-1}(\dot)$ is the generalized inverse function, and the probability density function such that:

$$ f_{adj}(y)=f[\!H^{-1}(y)]\frac{1}{Q[\!v(H^{-1}(y))]} $$

where *f*_*adj*_ is the density function of *T*_*adj*_ and *f* is the density function of *T*. Therefore, for an individual *i* on treatment *j*, the practicioners have the following measures, $\phantom {\dot {i}\!}E_{{adj}_{ji}}$, which is such that:

$$E_{{adj}_{ji}}={inf}_{i} \left[T_{{adj}_{ji}},\eta_{{adj}_{ji}} \right] $$

where $\phantom {\dot {i}\!}\eta _{{adj}_{ji}}$ represents the censoring adjusted on quality of life and $\phantom {\dot {i}\!}T_{{adj}_{ji}}$ the survival time having the same adjustment. Also, let *C*_*ji*_ the cumulative cost for individual *i* on arm *j*. Thus, we get the following dependency evidences:

1. $\phantom {\dot {i}\!}T_{{adj}_{ji}}$ and *η*_*ji*_ are dependent,
2. $\phantom {\dot {i}\!}T_{{adj}_{ji}}$ and $\phantom {\dot {i}\!}\eta _{{adj}_{ji}}$ are independent,
3. *C*_*ji*_ and *η*_*ji*_ are dependent.

Proofs are provided in the “Appendix” section.

#### Estimation of the parameters of the distributions

Even if, to begin, the right distributions for costs and QALY are unknown, it is possible to infer their two main parameters: mean and variance. In fact, we will consider here each arm of the trial as a distinct random variable with distinct mean and variance but with the same probability distribution. Furthermore, we will assume that non-administrative censoring exists as the main consideration of the estimation. Let $\boldsymbol {Z_{ji}^{C}}$ be the d-vector of covariates that affect costs for arm *j*, *j*=0,1, for the grouped population, and $\boldsymbol {Z_{ji}^{E}}$ be the one for QALY. Then, as proposed by Thompson and Nixon [13] and Stamey et al. [14], the costs mean function, on an arm *j*, is defined as:

$$\mu_{j}^{C} =\alpha_{0}+\alpha_{1} z_{1j}^{C}+\ldots+\alpha_{d} z_{dj}^{C}, $$

and, the QALY variable mean function given costs is defined as:

$$\mu_{j}^{T_{adj}}=\beta_{0}+\beta_{1} z_{1j}^{T_{adj}}+\ldots+\beta_{d} z_{dj}^{T_{adj}}. $$

As these models are, in fact, linear regression models with censoring on covariates, using the method of Lin [15], one can estimate the regression coefficients vector *α*_*C*_ by the sum over the *k* periods of time of interest $\hat {\alpha }_{C}=\sum _{k=1}^{K} \hat {\alpha }_{C_{k}}$ using an inverse probability weighting method (IPCW) such that, for an individual *i* belonging to arm *j*,

$$\hat{\alpha}_{C_{k}}=\left(\sum_{i=1}^{n} \frac{\delta_{jki}^{\star}}{\hat{G}\left(X_{jki}^{\star}\right)}Z_{j}^{C} \left(Z^{C}_{j}\right)^{t} \right)^{-1} \sum_{i=1}^{n} \frac{\delta_{jki}^{\star} C_{jki}}{\hat{G}\left(X_{jki}^{\star}\right)} Z_{j}^{C} $$

where $X_{jki}^{\star }=\min (X_{ji}, a_{k+1}), \hat {G}(\bullet)$ is the Kaplan-Meier estimator of  and, as described in “Background” section, *X*_*ji*_ is the minimum between time from randomization to death and time from randomization to censoring, and . The same approach is used to find *β*, the vector of regression coefficients for QALY. Thus, from the inference on coefficients, it is possible to determine the adjusted mean on survival.

In terms of variance, we propose the use of the result of Buckley and James [16] (see also Miller and Halpern [17]), which is a generalization of the IPCW techniques. Thus, the approximate variance of the cost distribution on a given arm is:

$$\hat{\sigma}^{2}_{C_{j}}=\frac{1}{\sum_{l=1}^{n}\delta_{l} -2}\sum_{i=1}^{n} \delta_{i}\left(\hat{e}^{0}_{i}-\frac{1}{\sum_{l=1}^{n} \delta_{l}} \sum_{j=1}^{n} \delta_{j} \hat{e}_{j}^{0} \right)^{2} $$

where $\hat {e}_{0}^{i}$ is an error term such that $\hat {e}_{0}^{i}=C_{i}-\boldsymbol {Z_{j}^{C}}\hat {\alpha }_{j}$. A similar approach is done for QALY.

#### Determination of the parametric distributions

To model costs, three common distributions are frequently used: Gamma, Normal and Lognormal distributions. Their parametrization is easily done given the mean and the variance of the distribution. Let *μ*_*C*_ be the mean and $\sigma ^{2}_{C}$ be the variance of costs for any clinical arm *j*. Then, the parametric distribution choice will be one of the following:

1. $C_{j} \sim Normal \left (\mu _{C_{j}}, \sigma ^{2}_{C_{j}}\right),$
2. $\phantom {\dot {i}\!}C_{j} \sim {Gamma} (\mu _{C_{j}}, \rho _{C_{j}}),$
3. $C_{j} \sim Lognormal \left (\nu _{C_{j}},\tau ^{2}_{C_{j}}\right),$

where *ν*_*C*_ and $\tau ^{2}_{C}$ are mean and variance of the log-costs, i.e. $\nu _{C}=2log(\mu _{C})-\frac {1}{2}log\left (\sigma ^{2}_{C}+\mu _{C}^{2}\right)$ and $\tau _{C}^{2}=log\left (\sigma _{C}^{2}+\mu _{C}^{2}\right)-2log(\mu _{C})$. Furthermore, *ρ*_*C*_ is the shape parameter of the Gamma distribution, which is such that $\rho _{C}=\mu _{C}^{2}/\sigma ^{2}_{C}$. Thus, once each modeling is achieved, a selection of the better parametric distribution has to be done using the deviance criteria. The best fit to the data corresponds to the distribution that has the lower deviance, which is minus two times the log-likelihood.

In the case of QALY, the choice may be any symmetric or skew-symmetric distribution. We propose two options here, but the classical way here is to consider only a gaussian distribution following the work of Thompson and Nixon [13]. One notes that, even if it may look strange to use a real defined function for a real positive distribution, the distribution of *T*_*adj*_ usually has a high mean with a low standard error such that the negative part of the fitted distribution is in fact negligible. Then, the proposed options are:

1. $T_{{adj}_{j}} \sim Normal\left (\mu _{T_{adj}}, \sigma ^{2}_{T_{adj}}\right),$
2. $\phantom {\dot {i}\!}T_{{adj}_{j}} \sim Gamma (\mu _{T_{adj}}, \rho _{T_{adj}}).$

### Inference on Kendall’s tau

To further compute the copula parameter for each tested copula in the selection process, one has to get the global dependence parameter: Kendall’s tau. The idea is that performing inference on Kendall’s tau instead of directly on the copula parameter for each tested copula leads to only one estimation process instead of as many inferences as the quantity of tested models. Surely, the maximum likelihood estimator can be used in order to get the copula parameter. However, in this paper, we suggest the use of Kendall’s tau for non-randomized data in reason of the easiness of the method. Furthermore, as shown by Genest et al. [18, 19], the difference between both inference methods is slightly significant compared to the gain in computational efficiency. Note that in case of randomized data, the exchangeability phenomenon among individuals occurs and concordance measure may be biased. Hence, in this case, we suggest the use of a standard maximum likelihood estimation for the copula parameter.

The Kendall’s tau inversion method to infer a copula parameter has been shown to be consistent under specific assumptions, which holds here [20]. In fact, for every parametric copula family, there exists a direct relationship between the copula parameter and the Kendall’s tau. For example, with Clayton copula, one has $\hat {\theta }=2\tau /(1-\tau)$ and for Gumbel copula, $\hat {\theta }=1/(1-\tau)$. These relationships are clearly given in almost all the literature about parametric copulas [12]. Let consider the couple of random variables (*C,T*_*adj*_) on a fixed therapeutic arm *j*, *j*=0,1. Furthermore, let consider $\left (C^{\{1\}},T_{{adj}}^{\{1\}}\right)$ and $\phantom {\dot {i}\!}\left (C^{\{2\}},T_{{adj}}^{\{2\}}\right)$ two independent joint observations of the couple (*C*,*T*_*adj*_). Then, the pair is said concordant if $\left (C^{\{1\}}-C^{\{2\}}\right)\left (T_{{adj}}^{\{1\}}-T_{{adj}}^{\{2\}}\right)>0$ and discordant elsewhere. The Kendall’s tau, which is in fact a concordance measure, is defined in Kendall [21] by  where $\mathbb {E}$ is the expectation,  is the indicator function,  and . In a more general frame, one has the couples $\left (C^{\{1\}},T_{adj}^{\{1\}}\right),\left (C^{\{2\}}, T_{adj}^{\{2\}}\right), \ldots,\left (C^{\{n\}}, T_{adj}^{\{n\}}\right)$ where all the values of $C^{\{r\}}, T_{adj}^{\{r\}}, r=1,\ldots,n$ are unique. Thus, one can write  and  where *r* and *s* are the index of the independent replications. In absence of censoring, the estimation of *τ* is given by its sample version:

$$\hat{\tau}_{K}=\binom {n}{2}^{-1}\sum_{1\leq r < s \leq n} a_{rs}b_{rs}, $$

where *n* is the sample size. In fact, it is simply the *n*(*n*−1)/2 pairs of bivariate observations that could be constructed, multiplied by the subtraction of the number of discordant pairs to the number of concordant pairs. Under censoring, the approach of Oakes [22] propose to add an uncensoring indicator  to that equation such that:

$$\tilde{\tau}_{K}=\binom {n}{2}^{-1}\sum_{1\leq r < s \leq n} L_{rs}a_{rs}b_{rs}, $$

where *U* ^{*r*}^ and *U* ^{*s*}^ represent the censoring variables under each independent replication. The problem with that estimator is the lack of consistency on a high-dependent scheme. Therefore, one advocates the use of the renormalized Oakes’ estimator [23] for which consistency has been shown. Therefore, the estimator

$$\hat{\tau}_{K}= \frac{\sum_{\{1\leq r < s \leq n\}} L_{rs}a_{rs}b_{rs}}{\sum_{\{1\leq r < s \leq n\}} L_{rs}} $$

is simply the ratio of the subtraction of the number of uncensored discordant pairs to the number of uncensored concordant pairs over the total of uncensored pairs.

#### Bayesian selection of the copula

Inference here may be done on any consistent parametric copula. To select the right copula, few alternatives are proposed in the literature. For complete data distributions, Genest and Rivest [18] proposed a procedure based on a probability integral transformation. Furthermore, based on the goodness-of-fit, Lakhal-Chaieb [24] proposed a procedure for censored data when the distributions are estimated by the survival functions. However, when available, a prior knowledge of the distribution of the margins in a copula is not a negligible information. It should be taken into account for the inference of the copula model when the latter is unknown, to minimize the risk of errors. In their paper, Dos Santos Silva et al. [25] proposed a method of selection based on the information criterion. Let note $F_{T_{adj}}(y)$ and *F*_*C*_(*χ*) the cumulative distribution functions for QALY and costs for a given randomization arm. Thus, one gets:

$${} \begin{aligned} f(y,\chi|\Phi)&= c(F_{T_{adj}}(y|\Phi_{T_{adj}}),F_{C}(\chi|\Phi_{C})|\Phi_{\theta})\\ &\quad\times f_{T_{adj}}(y|\Phi_{T_{adj}})f_{C}(\chi|\Phi_{C}) \end{aligned} $$

where *Φ*_*C*_ stands for a parameter vector comprising parameters of the distribution of costs, $\Phi _{T_{adj}}$ is the parameter vector for QALY distribution, *Φ*_*θ*_ is the dependence parameter which is functional of the Kendall’s tau and $\Phi =\Phi _{C} \cup \Phi _{T_{adj}} \cup \Phi _{\theta }$ is the union of all these vectors of parameters. Also, *f* and *F* respectively stand for the density probability function and the cumulative probability function. Note that a similar writing could be done for a multivariate modeling. As shown in Genest et al. [26], one can find the copula parameter of any parametric copula while just having the Kendall’s tau [21] measure, even in multivariate models [27].

Let ***x*** be a bivariate sample of size *n* of that density function. Also, let ${\mathcal {M}}_{k}$ a copula model for *k*=1..*m* where *m* is the quantity of models one wants to test. Therefore, the likelihood function is given by:

$${{\begin{aligned} &\!\! L\left(\boldsymbol{x}|\Phi,\mathcal{M}_{k}\right)=\prod_{j=1}^{n} \left[ c\left(F_{T_{adj}}\left(y_{j}|\Phi_{T_{adj}},\mathcal{M}_{k}\right),F_{C}\right.\right.\\ &\times\left.\left.\left(\chi_{j}|\Phi_{C},\mathcal{M}_{k}\right)\!|\!\Phi_{\theta},\mathcal{M}_{k}{\vphantom{F_{T_{adj}}(y_{j}|\Phi_{T_{adj}},\mathcal{M}_{k}),F_{C}}}\right) f_{T_{adj}}\!\left(y_{j}|\Phi_{T_{adj}},\mathcal{M}_{k}\!\right)\!f_{C}\!\left(\chi_{j}|\Phi_{C},\mathcal{M}_{k}\right)\right]^{\!\delta_{j}} \\ & \times \left[1\,-\,C\left(F_{T_{adj}}\left(y_{j}|\Phi_{T_{adj}},\mathcal{M}_{k}\right),F_{C}\left(\chi_{j}|\Phi_{C},\mathcal{M}_{k}\right)\!|\!\Phi_{\theta},\mathcal{M}_{k}\right)\!\right]^{1-\delta_{j}} \\ & \times \left[ c\left(F_{\eta_{adj}}\left(y_{j}|\Phi_{\eta_{adj}},\mathcal{M}_{k}\right),F_{C}\left(\chi_{j}|\Phi_{C},\mathcal{M}_{k}\right)|\Phi_{\theta},\mathcal{M}_{k}\right)\right.\\ &\times\left. f_{\eta_{adj}}\left(y_{j}|\Phi_{\eta_{adj}},\mathcal{M}_{k}\right)f_{C}\left(\chi_{j}|\Phi_{C},\mathcal{M}_{k}\right)\right]^{1-\delta_{j}} \\ & \times \left[1-C\left(F_{\eta_{adj}}\left(y_{j}|\Phi_{\eta_{adj}},\mathcal{M}_{k}\right),F_{C}\left(\chi_{j}|\Phi_{C},\mathcal{M}_{k}\right)|\Phi_{\theta},\mathcal{M}_{k}\right)\right]^{\delta_{j}}\!, \end{aligned}}} $$

where *δ*_*j*_ indicates if an individual is censored or not. Then, using the deviance function which is $D(\Phi _{k})=-2ll(\boldsymbol {x}|\Phi,\mathcal {M}_{k})$ where *ll* stands for the log-likelihood function, Dos Santos Silva et al. [25] proposed to use the deviance information criterion (DIC) which is:

$$\begin{array}{@{}rcl@{}} DIC(\mathcal{M}_{k})=2\mathbb{E}[\!D(\Phi_{k})|\boldsymbol{x},\mathcal{M}_{k}]-D(\mathbb{E}[\!\Phi_{k}|\boldsymbol{x},\mathcal{M}_{k}]). \end{array} $$

They proposed to use the Monte Carlo approximations to $\mathbb {E}[\!D(\Phi _{k})|\boldsymbol {x},\mathcal {M}_{k}]$ and $\mathbb {E}[\!\Phi _{k}|\boldsymbol {x},\mathcal {M}_{k}]$ which are respectively $L^{-1}\sum _{l=1}^{L} D\left (\Phi _{k}^{l}\right)$ and $L^{-1}\sum _{l=1}^{L} \Phi _{k}^{l}$. Here, one supposes that $\left \{ \Phi ^{(1)}_{k},\ldots,\Phi _{k}^{(L)} \right \}$ is a sample from the posterior distribution $f(\Phi _{k}|\boldsymbol {x},\mathcal {M}_{k})$. Then, one chooses the copula model in all the chosen range with the smaller DIC.

One remarks that such a selection process requests parametric copulas with a limited quantity of parameters. Hence, one suggests the use of archimedean and elliptic copulas families for these modelings. In fact, the range of dependance structure models provided by archimedean copulas is enough large to cover the dependance parametrisation in a cost-effectiveness analysis. For example, a Clayton copula may represent a study where a small QALY is directly linked to small costs, but a huge QALY is independent of costs. Also, one remarks that if costs and QALY are independents, then the independence copula is selected and one gets directly the values that constitute the ICER.

### Incremental cost-effectiveness ratio

From the estimated joint densities *f*(*y*_*j*_,*χ*_*j*_),*j*∈{0,1}, one writes, for costs:

$$\begin{array}{@{}rcl@{}} \mathbb{E}[\!C_{j}] &=& \int_{\mathbb{D}_{C_{j}}} \int_{\mathbb{D}_{T_{{adj}_{j}}}} \chi_{j} f\left(\chi_{j},y_{j}\right) {dy}_{j} d\chi_{j} \\ &\approx & \int_{\mathbb{D}_{C_{j}}} \int_{\mathbb{D}_{T_{{adj}_{j}}}} \chi_{j} c_{\hat{\theta}}^{(i)}\left(\tilde{F}_{T_{adj}}\left(y|\hat{\Phi}_{T_{adj}}\right),\tilde{F}_{C}\left(\chi|\hat{\Phi}_{C}\right)\right)\\ &&\times\tilde{f}_{C}\left(y|\hat{\Phi}_{C}\right) \tilde{f}_{T_{adj}}\left(\chi|\hat{\Phi}_{T_{adj}}\right) {dy}_{j} d\chi_{j} \end{array} $$

where $\mathbb {D}_{C_{j}}$ and $\mathbb {D}_{T_{{adj}_{j}}}$ are respectively the domain of definition for the random variables *C* and *T*_*adj*_ for arm *j*. For QALY, one has the following:

$$\begin{array}{@{}rcl@{}} \mathbb{E}[\!E_{j}]&=&\int_{\mathbb{D}_{T_{{adj}_{j}}}} \int_{\mathbb{D}_{C_{j}}} y_{j} f\left(\chi_{j},y_{j}\right) d\chi_{j} {dy}_{j} \\ &\approx & \int_{\mathbb{D}_{T_{{adj}_{j}}}} \int_{\mathbb{D}_{C_{j}}} y_{j} c_{\hat{\theta}}^{(i)}\left(\tilde{F}_{T_{adj}}\left(y|\hat{\Phi}_{T_{adj}}\right),\tilde{F}_{C}\left(\chi|\hat{\Phi}_{C}\right)\right)\\ &&\times\tilde{f}_{C}\left(y|\hat{\Phi}_{c}\right) \tilde{f}_{T_{adj}}\left(\chi|\hat{\Phi}_{T_{adj}}\right) d\chi_{j} {dy}_{j}. \end{array} $$

Thus, given the expected costs and survival time adjusted to quality of life, the adjusted ICER is estimated by

$$\widehat{ICER}=\frac{\widehat{\mathbb{E}(C_{j=1})}-\widehat{\mathbb{E}(C_{j=0})}} {\widehat{\mathbb{E}(T_{{adj}_{j=1}})}-\widehat{\mathbb{E}(E_{{adj}_{j=0}})}} $$

and using Fieller’s theorem (Fieller [28], Willan and O’Brien [29], Chaudhary and Stearns [30]), one gets the 100(1−*α*)*%* confidence interval such that

$$\widehat{ICER}\left(\frac{\left(1-z^{2}_{1-\alpha /2} \hat{\sigma}_{\Delta_{C}\Delta_{T_{adj}}} \right) \pm z_{1-\alpha /2} \sqrt{\hat{\sigma}^{2}_{\Delta_{T_{adj}}}+\hat{\sigma}^{2}_{\Delta_{C}}-2\hat{\sigma}^{2}_{\Delta_{C} \Delta_{T_{adj}}} -z^{2}_{1-\alpha /2}\big(\hat{\sigma}^{2}_{\Delta_{T_{adj}}}\hat{\sigma}^{2}_{\Delta_{C}}-\hat{\sigma}^{2}_{\Delta_{C} \Delta_{T_{adj}}}\big) }}{1-z^{2}_{1-\alpha /2}\hat{\sigma}^{2}_{\Delta_{T_{adj}}}}\right). $$

In this formula, *z*_1−*α*/2_ represents the 100(1−*α*/2) percentile of the standard normal distribution. Furthermore, $\hat {\sigma }^{2}_{\Delta _{T_{adj}}}$ represents the variance of the distribution for effectiveness where $\Delta _{T_{adj}}$ is the difference between $\phantom {\dot {i}\!}T_{{adj}_{j=1}}$ and $\phantom {\dot {i}\!}T_{{adj}_{j=0}}$. The same scheme arises for $\hat {\sigma }^{2}_{\Delta _{C}}$. For $\hat {\sigma }_{\Delta _{C} \Delta _{T_{adj}}}$, it is nothing more than the estimated covariance of the differences, between *j*=0 and *j*=1, in costs and in quality adjusted life years.

The reason to use Fieller’s theorem is to avoid standard ways of using bootstrap. As shown by Siani and Moatti [31], Fieller’s method is often as robust as bootstrap methods are (both parametric and non-parametric ways), even in problematic cases. However, for non-common situations, an approach based on the ICER graphical plan (as proposed by Bang and Zhao [32]) is recommended. Indeed, in such a case, a graphical approach minimize the use of biased or non-sufficient statistics in a parametric confidence interval model.

### Incremental net benefit

The adjusted INB(*λ*) is estimated by $\widehat {INB}=\lambda (\widehat {\mathbb {E}(T_{{adj}_{j=1}})}-\widehat {\mathbb {E}(T_{{adj}_{j=0}})})-(\widehat {\mathbb {E}(C_{j=1})}-\widehat {\mathbb {E}(C_{j=0})})$ with variance $\hat {\sigma }^{2}_{\lambda }=\lambda ^{2}\hat {\sigma }^{2}_{\Delta _{T_{adj}}}+\hat {\sigma }^{2}_{\Delta _{C}}-2\lambda \hat {\sigma }_{\Delta _{C} \Delta _{T_{adj}}}$ where *λ* is the willingness-to-pay for a unit of effectiveness.

### Subgroups analysis

It is possible to accomplish a cohort analysis using that procedure. The main idea here is to perform a cost-effectiveness analysis while achieving a discrimination between two or more subgroups. The principle is that there exists a baseline variable *Z*_*k*_,*k*∈{1,2,…,*d*}, even for costs than for QALY, which is in fact a categorical variable (dichotomous or multichotomous) and for which one should determine a marginal *INB*. Such subgroups have to be based on clinically important covariates. Since that these subgroups are in the therapeutic arms, it is not possible to assume that they are balanced. As shown in Nixon and Thompson [33], and Tsai and Peace [34], a naive use of these subgroups information without any adjustment may lead to serious bias.

Let $Z_{jki}^{C}, Z_{jki}^{T_{adj}}$ be some population attribute indicator covariates (e.g. sex, smoking status, etc.) for costs and QALY on an individual *i* belonging to the clinical arm *j*. For the sake of illustrating the concept here, let say that one tests the therapeutic effect on smokers. Therefore, there will be four subgroups: smokers in the treated group, non-smokers in the treated group, and the same for the control groups.

Let $\phantom {\dot {i}\!}T_{{adj}_{j=1,k=1,i}}$ be the effectiveness for smokers individuals *i* on the treated arm, $\phantom {\dot {i}\!}T_{{adj}_{j=1,k=0,i}}$ be the effectiveness for non-smokers individuals *i* on the treated arm; and the same for the control arm. One does a similar writing for costs. Let $\mathbb {E}(\overline {T}_{{adj}_{j}})=\mathbb {E}(T_{{adj}_{j,k=1}})-\mathbb {E}(T_{{adj}_{j,k=0}})$ and the same for costs. Then, the interest in this discrimination is on the incremental net benefit marginalized to the smokers cohort, which is $\overline {INB}(\lambda)=\lambda (\mathbb {E}(\overline {T}_{{adj}_{j=1}})-\mathbb {E}(\overline {T}_{{adj}_{j=0}}))-(\mathbb {E}(\overline {C}_{j=1})-\mathbb {E}(\overline {C}_{j=0}))$. Since that subgroups are inside clinical arms, the main issue is to establish the expression of variance. Adjusting Fieller’s method to the subgroups context, one has

$$\begin{array}{@{}rcl@{}} \mathbb{V}ar(\overline{INB}(\lambda)) &=& \lambda^{2}\sigma^{2}_{\Delta_{\overline{T_{adj}}}}+\sigma^{2}_{\Delta_{\overline{C}}}-2\lambda\sigma_{\Delta_{\overline{T_{adj}}} \Delta_{\overline{C}}} \\ &=& \lambda^{2} \mathbb{V}ar\left(\overline{T}_{{adj}_{j=1}}-\overline{T}_{{adj}_{j=0}}\right)\\ &&+\mathbb{V}ar\left(\overline{C}_{j=1}-\overline{C}_{j=0}\right) \\ &&- 2\lambda cov\left(\overline{T}_{{adj}_{j=1}}-\overline{T}_{{adj}_{j=0}},\overline{C}_{j=1}-\overline{C}_{j=0}\right) \end{array} $$

where variances are computed in the standard path. For the covariance term, $\sigma _{\Delta _{\overline {T_{adj}}} \Delta _{\overline {C}}}$, there are two possible scenarios. Firstly, when the assumption that subgroups in treated and control arms are randomized is possible, one has

$$\begin{aligned} &cov\left(\overline{T}_{{adj}_{j=1}}-\overline{T}_{{adj}_{j=0}},\overline{C}_{j=1}-\overline{C}_{j=0}\right)\\ &\qquad\quad= cov\left(\overline{T}_{{adj}_{j=1}}, \overline{C}_{j=1}\right) + cov\left(\overline{T}_{{adj}_{j=0}}, \overline{C}_{j=0}\right) \end{aligned} $$

which can be found easily using standard techniques. Secondly, when the randomization assumption between subgroup is not possible because the cohorts are unbalanced in the clinical arms, then the covariance is:

$$\begin{array}{@{}rcl@{}} cov(\overline{T}_{{adj}_{j=1}}&-&\overline{T}_{{adj}_{j=0}},\overline{C}_{j=1}-\overline{C}_{j=0})\\ =&& cov\left(\overline{T}_{{adj}_{j=1}}, \overline{C}_{j=1}\right)+ cov\left(\overline{T}_{{adj}_{j=0}}, \overline{C}_{j=0}\right)\\ &-& cov\left(\overline{T}_{{adj}_{j=1}}, \overline{C}_{j=0}\right)-cov\left(\overline{T}_{{adj}_{j=0}}, \overline{C}_{j=1}\right). \end{array} $$

Here, the terms $cov(\overline {T}_{{adj}_{j=1}}, \overline {C}_{j=1})$ and $cov(\overline {T}_{{adj}_{j=0}}, \overline {C}_{j=0})$ are computed in a standard way similarly to the randomized case. However, for the crossed-arms covariance terms, $cov(\overline {T}_{{adj}_{j=1}}, \overline {C}_{j=0})$ and $cov(\overline {T}_{{adj}_{j=0}}, \overline {C}_{j=1})$, the approach that we suggest is to estimate the cumulative joint distributions $F(\overline {E}_{j=1}, \overline {C}_{j=0})$ by $C_{\hat {\theta }}(\hat {F}_{\bar {E}}(y_{j=1}),\hat {F}_{\bar {C}}(\chi _{j=0}))$ and $F(\overline {T}_{{adj}_{j=1}}, \overline {C}_{j=0})$ by $C_{\hat {\theta }}(\hat {F}_{\bar {T_{adj}}}(y_{j=1}),\hat {F}_{\bar {C}}(\chi _{j=0}))$ and *F* $(\overline {T}_{{adj}_{j=1}}, \overline {C}_{j=0})$ by $C_{\hat {\theta }}(\hat {F}_{\bar {T_{adj}}}(y_{j=1}),\hat {F}_{\bar {C}}(\chi _{j=0}))$ according to the methodology shown in this paper, and then using the covariance definition $cov(\overline {C},\overline {T_{adj}})=\mathbb {E}[\!\overline {T_{adj}},\overline {C}]- \mathbb {E}[\!\overline {T_{adj}}]\mathbb {E}[\overline {C}]$, compute the desired covariance from the estimated joint density function and the estimated marginal density functions.

In the spirit of the test of Willan et al. [6], one can test the equality of the INB between cohorts, which can be rejected at a level *α* if

$$\frac{\left|\overline{INB}(\lambda)\right|}{\sqrt{\mathbb{V}ar(\overline{INB}(\lambda))}} $$

is greater than the *z*_1−*α*/2_ percentile of a standard gaussian distribution.

## Results and discussion

This section gives an illustration of the performance of the copula which provides the best estimate of the true copula and the cumulative joint distribution of costs and QALY respectively, according to the method presented above. Let the exact copula be $C_{\theta }^{(\star)}(F_{T_{adj}}(y|\Phi _{T_{adj}}),F_{C}(\chi |\Phi _{C}))$ and its estimate be $C_{\hat {\theta }}^{(i)}(\tilde {F}_{T_{adj}}(y|\hat {\Phi }_{T_{adj}}),\tilde {F}_{C}(\chi |\hat {\Phi }_{C}))$ where (*i*) is the copula model selected among all the tested models and (⋆) the exact copula model. Furthermore, $\tilde {F}$ is the chosen distribution for *F*. Then, the objective of these simulations is to show that the bias generated by the approximation of *θ* by $\hat {\theta }, \Phi =\Phi _{C} \cup \Phi _{T_{adj}}$ by $\hat {\Phi }$, the selection of *C* ^(*i*)^ as the copula model and $\tilde {F}_{C}, \tilde {F}_{T_{adj}}$ as the marginal parametric models, is relatively weak.

In order to evaluate the performance of the proposed method in non-trivial cases, we performed Monte-Carlo simulations on 27 different simulation schemes. The methodology was to simulate bivariate data (representing costs and QALY) from three specific copulas. For each copula, we simulated the three possible configurations for the marginal distributions (costs are either normally distribute or lognormally distributed or follow a gamma distribution, while QALY is normally distributed). Then, we applied three different levels of randomly censoring on the marginal distribution of QALY (15,30 and 70%). Hence, there were nine possibles copulas configurations; for these nine, we also challenged our methodology without any censoring to get a point of comparison for the inference of the Kendall’s tau part of the model. For all the simulations, we assumed that *T*_*adj*_ follows a normal distribution. Alternatively, we could have set *T*_*adj*_ following a Gamma distribution. However, in the present core, the goal relatively to margins was to check that the determination of the marginal distributions criterion was adequate, which may be tested on only one margin (to simplify the text). The censoring followed an exponential distribution such that *λ*_*s*=15_=0.041,*λ*_*s*=30_=0.090 and *λ*_*s*=70_=0.308 where *s* represents the censoring percentage simulated. For all data generating processes (DGP), the Kendall’s tau was identical and represented an intermediate level of dependence between marginal distributions to be fair with the reality: *τ*_*K*_=0.60. Then, we computed the right copula parameter, for each copula, based on this Kendall’s tau. We also used a relatively standard mean and variance in cost-effectiveness analysis, following parameters used by Thompson and Nixon [13], such that *μ*_*C*_=1500,*σ*_*C*_=400; $\mu _{T_{adj}}=4, \sigma _{T_{adj}}=0.75$, and we parametrized each marginal distribution to keep close to these values. For the choice of generating copulas, we selected the three most-known ones with the biggest difference: Gaussian, Clayton and Gumbel copulas. The DGPs schemes is shown on Table 1.

**Table 1:** Scheme of the 27 data generated cases

Generating copula	Costs distribution	Censoring level	DGP
Gaussian copula	$F_{C} \sim \mathcal {N}(\mu _{C}=1500, \sigma _{C}=400)$	15%	DGP 1
*θ*≈0.809		30%	DGP 2
		70%	DGP 3
	*F*_*C*_∼*Γ*(*shape*_*C*_=12,*scale*_*C*_=125)	15%	DGP 4
		30%	DGP 5
		70%	DGP 6
	$F_{C} \sim log\mathcal {N}(\nu _{C}=7.30,\tau _{C}=0.25)$	15%	DGP 7
		30%	DGP 8
		70%	DGP 9
Clayton copula	$F_{C} \sim \mathcal {N}(\mu _{C}=1500, \sigma _{C}=400)$	15%	DGP 10
*θ*=3		30%	DGP 11
		70%	DGP 12
	*F*_*C*_∼*Γ*(*shape*_*C*_=12,*scale*_*C*_=125)	15%	DGP 13
		30%	DGP 14
		70%	DGP 15
	$F_{C} \sim log\mathcal {N}(\nu _{C}=7.30,\tau _{C}=0.25)$	15%	DGP 16
		30%	DGP 17
		70%	DGP 18
Gumbel copula	$F_{C} \sim \mathcal {N}(\mu _{C}=1500, \sigma _{C}=400)$	15%	DGP 19
*θ*≈0.809		30%	DGP 20
		70%	DGP 21
	*F*_*C*_∼*Γ*(*shape*_*C*_=12,*scale*_*C*_=125)	15%	DGP 22
		30%	DGP 23
		70%	DGP 24
	$F_{C} \sim log\mathcal {N}(\nu _{C}=7.30,\tau _{C}=0.25)$	15%	DGP 25
		30%	DGP 26
		70%	DGP 27

The simulation of linearly dependent and uncensored covariates for costs leads to a bias in our advantage for the computation of the mean and the variance, compared to the estimation performed according to a standard clinical scheme. We decided therefore to challenge our method using the Kaplan-Meier mean estimate of the survival function and its associated variance (which is the appropriate approach in absence of covariates of interest) instead of the presented procedure based on the covariates. Then, we applied the following steps: selecting a parametric distribution for the margins, selecting a parametric copula using the information criterion and finally, looking for the copula parameter. We replicated this procedure 500 times for *n*=1000 data, then we collected the provided information on the frequency of successful procedures for the inference on the margins, the estimated Kendall’s tau and the choice of copula, respectively.

***Inference on Kendall’s tau***

**Fig. 1:**
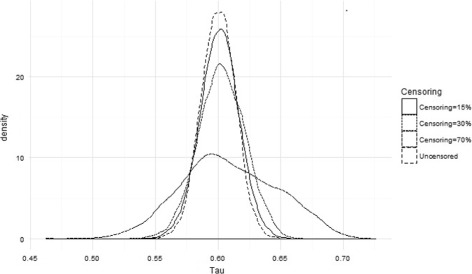
Inference on Kendall’s tau according to the simulations. Dispersion of the estimated Kendall’s tau according to the censoring level around the theoretical value of tau used to generate data: 0.6

The proposed way to infer Kendall’s tau under censoring gives results close to the real *τ*_*K*_ measured on data just before applying the censoring. On Fig. 1 and Table 2, one can see the dispersion of the computed Kendall’s tau. We remind the reader that the theoretical value of tau used for simulations is 0.6, and that we performed simulations without censoring to give an idea of the Kendall’s tau measure on complete data. Thus, firstly, all simulations were performed without any censoring. Secondly, a 15% rate of censoring were applied on data. Thirdly, for all the simulations, we used a 30% rate of censoring and, for the last case, we applied a 70% rate of censoring. Therefore, the vector that contains all the inferred Kendall’s tau for a (un)censoring level has a length of 4500. One observes that the estimated values of $\hat {\tau _{K}}$ are relatively closes to the theoretical value of 0.6, but one remarks that the dispersion of values for some $\hat {\theta }$ is not close to the expected value as is the dispersion on $\hat {\tau }_{K}$, since *τ*_*K*_ ranges in [−1,1] while, for example, for Clayton copula, *θ* ranges in [−1,*∞*)∖{0}.

**Table 2:** Information on the estimation of the Kendall’s tau for each censoring level

Censoring level	Mean $(\hat {\tau }_{K})$	Var $(\hat {\tau }_{K})$	Min $(\hat {\tau }_{K})$	Max $(\hat {\tau }_{K})$
Censoring =0%	0.6002	0.00019	0.5488	0.6476
Censoring =15%	0.6011	0.00024	0.5416	0.6539
Censoring =30%	0.6030	0.00035	0.5319	0.6648
Censoring =70%	0.6089	0.00146	0.4624	0.7257

The outputs of the 9 simulations with a censoring of 0% are joint together in the information on the first line and the same for simulations censored at 15% on the second line, 30% at the third line and 70% at the last line

***Inference on the marginal distribution for the costs***

**Fig. 2:**
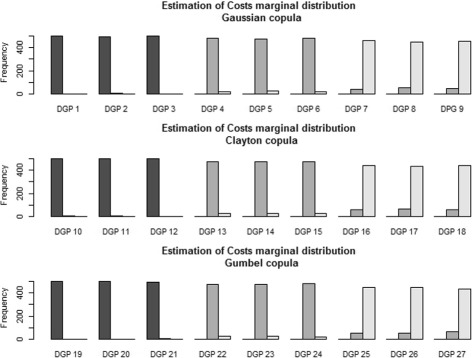
Frequency of selection of parametric marginal distributions for costs from the deviance criteria for each data generating process (DGP). The *black bar* represents the selection of the Normal distribution, the *dark-gray bar* represents the selection of the Gamma distribution and the *light-gray* stands for a logNormal distribution

To select the right marginal distribution for costs on each of the 500 simulations and for each DGP, we used the proposed criteria based on the deviance. Thus, on Fig. 2, one can see the performance of this criterion. One may remark that, even with a 70% rate of censoring, the chosen parametric distribution is almost always correctly estimated.

***Inference on the copula models***

There exists a bunch of parametric copula families, but for the purpose of these simulations, we limited our selection to the most well-known ones: Gaussian, Student, Clayton, Gumbel, Frank and Joe copulas. However, with a real dataset, the reader may test any consistent parametric copula using the proposed way. During simulations, for each iteration, we collected the information about the selected family using the information criterion. At Tables 3 and 4, one can see these results for each DGP. We indicated, in bold, the copula that was selected the most in the 500 iterations and claimed that it was the copula to be retained for the mentioned DGP. When the selection of the copula was only in-between those used to simulate the data (as on Table 3), it was obvious that the chosen copula family is the one used for the generation process. Otherwise, when intermediate copulas (for which dependence adequation is close to the one provoked by the generating copula) are introduced in the selection process, the results may differ as shown on Table 4. For the DGPs where the costs were simulated following a normal distribution (1,2,3,10,11,12,19,20 and 21), the choice of the copula was influenced by the dependence caused by the type of copula retained to generate the data. In facts, when the costs margin follows $\mathcal {N}(\mu _{C}=1500, \sigma _{C}=400)$ and the QALY margin follows $\mathcal {N}(\mu _{T_{adj}}=4, \sigma _{T_{adj}}=0.75)$, using *τ*_*K*_=0.60, if the dependency distribution is mostly normal (i.e. comes from a Gaussian or a Gumbel copula), there will be a moderate tail on both tails of the distribution with an uniform cloud along the correlation path. For these reasons which are attributes of the Frank copula, it was the chosen copula for DGPs 1,2,3,19,20 and 21. However, when, from the generating Clayton copula, a strict dependence was imposed to the left-tail while a mostly total independence was imposed to the right-tail, only a Clayton copula appeared appropriate to modeling these data. That’s the reason why it was the chosen copula for DGPs 10,11 and 12. In the situation where QALY follows $\mathcal {N}(\mu _{E}=4, \sigma _{E}=0.75)$ while costs follows a skewed marginal distribution (gamma or lognormal), in any case, there was not a right-tailed distribution, but mostly a left-oriented data cloud with a fat left-tail. That is the reason why, even when the data were generated from a Clayton copula, the Student (t) copula was the chosen one. Therefore, the simulations showed that the Bayesian criterion of selection of the copula was in accordance with the theoretical properties of the parametric copulas.

**Table 3:** Frequency of choice of a copula for each DGP given 500 iterations for the three main copulas

DGP	Gaussian	Clayton	Gumbel
	copula	copula	copula
DPG 1	**460**	0	40
DGP 2	**454**	0	46
DGP 3	25	94	**381**
DGP 4	**415**	0	85
DGP 5	**429**	0	71
DGP 6	140	21	**339**
DGP 7	**473**	0	27
DGP 8	**490**	0	10
DGP 9	**261**	10	229
DGP 10	0	**500**	0
DGP 11	0	**500**	0
DGP 12	0	**499**	1
DGP 13	18	**482**	0
DGP 14	11	**489**	0
DGP 15	68	**371**	61
DGP 16	2	**498**	0
DGP 17	6	**493**	1
DGP 18	177	**531**	62
DGP 19	2	0	**498**
DGP 20	9	0	**491**
DGP 21	1	9	**490**
DGP 22	123	0	**377**
DGP 23	109	0	**391**
DGP 24	36	0	**467**
DGP 25	80	0	**420**
DGP 26	84	0	**416**
DGP 27	121	0	**379**

The chosen copula is in bold font

**Table 4:** Frequency of choice of copula for each DGP on 500 iterations beyond the three main copulas and intermediate copulas

DGP	Gaussian	Student	Clayton	Gumbel	Frank	Joe
	copula	copula	copula	copula	copula	copula
DPG 1	7	45	6	25	**415**	2
DGP 2	2	44	4	25	**425**	1
DGP 3	4	54	4	17	**421**	0
DGP 4	32	**297**	0	56	112	3
DGP 5	26	**291**	0	59	120	4
DGP 6	23	**284**	0	79	113	1
DGP 7	47	**343**	1	45	38	26
DGP 8	55	**344**	0	43	33	25
DGP 9	55	**328**	0	56	37	24
DGP 10	0	12	**364**	0	124	0
DGP 11	0	17	**372**	0	111	0
DGP 12	0	5	**385**	0	110	0
DGP 13	0	**427**	56	0	17	0
DGP 14	2	**433**	55	0	10	0
DGP 15	1	**431**	58	0	10	0
DGP 16	1	**477**	19	2	1	0
DGP 17	2	**476**	18	0	4	0
DGP 18	4	**472**	19	0	4	1
DGP 19	0	18	0	110	**196**	176
DGP 20	0	20	0	107	**214**	159
DGP 21	0	17	0	109	**202**	172
DGP 22	13	**236**	0	153	67	31
DGP 23	19	**231**	0	157	58	35
DGP 24	23	161	0	**204**	75	37
DGP 25	32	**198**	0	177	13	80
DGP 26	39	**213**	0	148	13	87
DGP 27	37	**210**	0	167	17	69

The chosen copula is in bold font

According to the structure of the marginal distributions, the choice of the copula could only be done in a limited spectrum of families. Therefore, when one seeks to find the best structural copula family, it appears essential to include the most known copula families covering as many dependence states as possible. Thus, in harmony with Table 4, a selected copula which is not the generating one is only performing a better adequacy to the dependence between margins structures than the original one.

One remarks that an high censoring level does not really impact the issue of the copula estimation. Indeed, for all DGPs with 70% censoring level, there is only one case where the result changes: DGP 24. In fact, the Gumbel copula is left-skewed, as Student copula may be; which can explains the wrong estimation of the copula in this case.

### Example: acupuncture for chronic headache in primary care data

We used the acupuncture for chronic headache in primary care data from Vickers et al. [35–37] containing migraine and chronic tension headache of 401 patients aged 18 to 65 years old who reported an average of at least two headaches per month. Subjects were recruited in the general practice context in England and Wales and they were allocated to receive until 12 acupuncture treatments for a period of three months. For the sake of the study, acupuncture intervention was provided in the community by the United Kingdom National Health Service (NHS). The study starts in 2002 with a time horizon of 12 months and was registered ISRCTN96537534.

The data collection focuses on the measure of effectiveness in terms of QALY gained and the cumulative cost associated in UK pounds (£). Patients themselves reported unit costs associated with non-prescription drugs and private healthcare visits. The cost of the study intervention was estimated from the standard cost for a NHS professional multiplied by the contact time with the patient. Thus, patients in the treated arm had a mean time of 4.2 hours with study acupuncturist. No imputation for missing data was done if the three questionnaires were not complete and consequently for which QALY cannot be measured. Therefore, in the acupuncture arm, there was 136 participants and in the control arm, 119. The modeling process of both joint distributions function for QALY and costs in the two clinical arms is presented on Table 5. We remark that in both cases, dependence is weak since that Kendall’s tau ranges between -0.10 and -0.15. Here, for the distribution of *T*_*adj*_, we compared two possibles choices: a Gamma distribution and a gaussian one. The normal distribution has the smallest deviance. For the choice of distributions for costs, in the two arms, the lognormal distribution was considered as the one with the smallest deviance. For the copula family selection using deviance information criteria, we compared, for each arm, Gaussian, Clayton, Student, Frank, Joe and Gumbel copulas. In the treated arm, Student copula was the considered one while in the control arm, it was the Gaussian copula. Therefore, the joint distribution function of the acupuncture arm was estimated by:

$${{\begin{aligned} \hat{F}\left(C_{j=1},T_{{adj}_{j=1}}\right)&=C_{\hat{\theta}=-0.1923}^{\left(Student\right)}\\ &\quad\times \left({\vphantom{\left(\!\hat{\mu}_{T_{adj}}=0.7268, \hat{\sigma}_{T_{adj}}=0.1190\right)}}F_{C} \sim logN\left(\hat{\nu}_{C}=5.7111, \hat{\tau}_{C}=0.7600\right),\right.\\ &\quad\left. F_{T_{adj}} \sim \mathcal{N}\left(\!\hat{\mu}_{T_{adj}}=0.7268, \hat{\sigma}_{T_{adj}}=0.1190\right)\!\right) \end{aligned}}} $$

while, for the control arm, the estimation is:

$${{\begin{aligned} \hat{F}\left(\!C_{j=0},T_{{adj}_{j=0}}\!\right)&=C_{\hat{\theta}=-0.1664}^{\left(Gaussian\right)}\\ &\quad\times\left(F_{C} \sim logN\left(\hat{\nu}_{C}=4.4844, \hat{\tau}_{C}=1.3390\right)\right.,\\ & \quad\left. F_{T_{adj}} \sim \mathcal{N}\left(\!\hat{\mu}_{T_{adj}}=0.7083, \hat{\sigma}_{T_{adj}}=0.1118\right)\!\right)\!. \end{aligned}}} $$

**Table 5:** Information gained in the analysis process for costs and QALY in both arms

Modelisation process	Control arm	Acupuncture arm
Kendall’s tau $(\hat {\tau }_{K})$	−0.1065	−0.1232
QALY distribution	$T_{adj} \sim \mathcal {N}(\hat {\mu }_{T_{adj}}=0.7083, \hat {\sigma }_{T_{adj}}=0.1118)$	$T_{adj} \sim \mathcal {N}(\hat {\mu }_{T_{adj}}=0.7268, \hat {\sigma }_{T_{adj}}=0.1190)$
Costs statistics	$\hat {\mu }_{C}=217.20$	$\hat {\mu }_{C}=403.40 $
	$\hat {\sigma }_{C}=486.00$	$\hat {\sigma }_{C}=356.59$
Costs distribution	$C \sim logN(\hat {\nu }_{C}=4.4844, \hat {\tau }_{C}=1.3390)$	$C \sim logN(\hat {\nu }_{C}=5.7111, \hat {\tau }_{C}=0.7600)$
Selected copula family	Gaussian	Student (t)
Copula parameter $(\hat {\theta })$	−0.1664	−0.1923

Using the approach given from the copula densities, the ICER is estimated such that $\hat {ICER}=10082.68$£/ unit of effectiveness, with a confidence interval which is:

$${{\begin{aligned} \hat{ICER}\left(\frac{1+12.44z_{1-\alpha}^{2}\pm z_{1-\alpha /2} \sqrt{365336.81-9744.36\times z^{2}_{1-\alpha /2}} }{1-z^{2}_{1-0.0266\alpha /2}}\right) \end{aligned}}} $$

where *z*_1−*α*_ is the 100(1−*α*/2) percentile of the standard gaussian distribution. It means that it costs approximately 10082.68 per year to get an additional unit of effectiveness using acupuncture for headache.

**Fig. 3:**
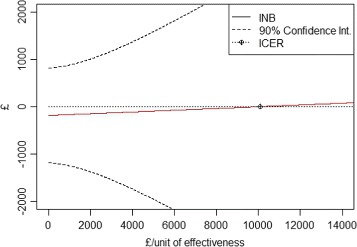
Plot of INB versus *λ* for acupuncture for headaches in primary care example. *λ* stands for the willingness-to-pay for a unit of effectiveness

The plot of the estimated INB with his 90 percent confidence limits versus lambda in presented on Fig. 3. The vertical intercept shows the negative value of the variability of costs and its confidence interval while the horizontal intercept shows the estimated ICER. Since the number of covariates was limited, it has not been possible to determine the existence of subgroups. Whether available, the search for subgroups is possible using our approach.

#### Impact of an artificially created censoring on the acupuncture example

To challenge our methodology via a censored dataset, we decided to create an artificial censoring on QALY variable (since that costs are assumed to always be observed). This censoring variable followed an exponential distribution where *λ*=0.30. Hence, the QALY variable has been censored on approximately 30% of data. Using the same methodology than for the acupuncture original dataset, we obtained the information shown on Table 6. One notes that we did not report the costs information on this table since that it does not change from the information on Table 5 (costs is not a censored variable).Using these information, one obtains an estimated ICER such that $\hat {ICER}=10879.89$£/ unit of effectiveness, with a confidence interval which is:

$${{\begin{aligned} \hat{ICER}\left(\!\!\frac{1+11.33z_{1-\alpha}^{2}\pm z_{1-\alpha /2} \sqrt{360533.67-7443.98\times z^{2}_{1-\alpha /2}} }{1-z^{2}_{1-0.0206\alpha /2}}\!\right)\!. \end{aligned}}} $$

**Table 6:** Information gained in the analysis process QALY in both arms, where an artificial censoring around 30 percents has been created

Modelisation process	Control arm	Acupuncture arm
Kendall’s tau $(\hat {\tau }_{K})$	−0.1388	−0.1011
QALY distribution	$T_{adj} \sim \mathcal {N}(\hat {\mu }_{T_{adj}}=0.7133, \hat {\sigma }_{T_{adj}}=0.1026)$	$T_{adj} \sim \mathcal {N}(\hat {\mu }_{T_{adj}}=0.7304, \hat {\sigma }_{T_{adj}}=0.1005)$
Selected copula family	Gaussian	Gaussian
Copula parameter $(\hat {\theta })$	−0.2163	−0.1582

Hence, with a certain level of censoring, the estimation of ICER loses accuracy, and the confidence interval gets larger. However, the estimated ICER in case of censoring stay relatively close to the estimated ICER with original data, and stays in his confidence interval.

#### Synthesis on the acupuncture example

Firstly, let take a look at the conclusions of Wonderling et al. [36], who were the first to work on these original data. Without totally detailing their methodology, they used a linear regression adjusted on covariates of interest (age, sex, diagnosis, severity of headache at baseline, number of years of headache disorder, baseline SF-36 results and geographical site) to evaluate the mean difference for costs and effectiveness (in terms of QALY). Hence, from a linear model, they have got an $\hat {ICER}$ equals to 9180 £with a mean health gain for acupuncture treatment of 0.021 QALY.

Using the copula-based methodology presented in this paper, we get, with these original data, a mean health gain for acupuncture treatment of 0.026 QALY and when we apply an exponentially distributed censoring around 30% on QALY variable, we get a mean health gain for acupuncture treatment of 0.021 QALY. The major difference between both approaches is on the estimated ICER value. However, we remark that the value of 9180 £keeps in the confidence interval of the copula-based ICER.

## Conclusion

One motivation for this work was generated by the limitations of the at standard regression models applied to the cost-effectiveness analysis where the dependence structure between costs and utility along with time was not taken into account. We provided a simple step-by-step procedure to find INB and ICER and their confidence intervals, even in case of censoring.

**Fig. 4:**
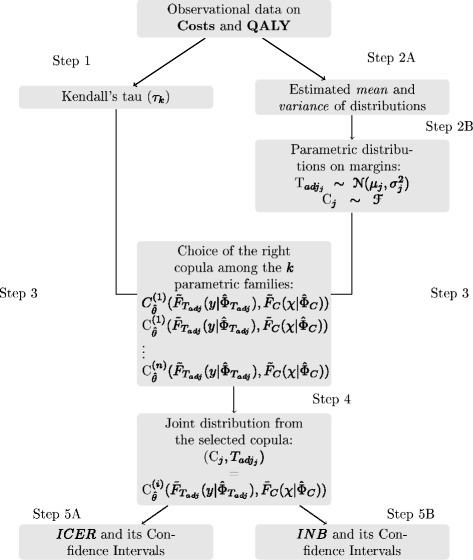
Schema of the procedure to perform cost-effectiveness analysis using copulas as shown in this paper

On Fig. 4, one sees the schematized method from the observational data produced by both clinical arms to the complete cost-effectiveness analysis. In a parallel way, one accomplishes steps 1 and 2 which are the measure of the dependence between QALY and cumulative costs in each arm via Kendall’s tau and the determination of the marginal distributions of both random variables. Then, at step 3, one generates copulas from information gained in steps 1 and 2 and, using the information criterion, one selects the closest copula to the right joint distribution function at step 4. Finally, at step 5, one determines the INB and the ICER using joint cdf. In case of subgroups cohorts analysis, one reiterate the procedure from step 1 to 5 to get two supplementary copulas and then, the joint cdf of costs and QALY for the crossed-arms covariance terms.

The methodology presented here can easily be implemented on computational software. Indeed, on R, using the packages copula [38] and CDVine [39], one can easily apply the whole process to a dataset, either censored or not. On SAS, the use of a PROC COPULA will be enough to fit a certain copula on a whole dataset.

## Appendix

### **Proposition 1**

The random variables $\phantom {\dot {i}\!}T_{{adj}_{ji}}$ and *η*_*ji*_ are dependent.

### *Proof*

To simplify the notation, let assume the following being for an individual *i* on a therapeutic *j*. Also, let $T_{adj}(\omega)=\int _{0}^{T(\omega)} Q(t) dt$. Given the observed times *E*(*ω*)= inf(*η*(*ω*),*T*(*ω*)). If *η*(*ω*)≤*T*(*ω*), one gets

$$\begin{array}{@{}rcl@{}} T_{adj}(\omega) &=& \int_{0}^{\eta(\omega)} Q(t)dt + \int_{\eta(\omega)}^{T(\omega)} Q(t)dt \\ &=& \eta_{adj}(\omega)+ \int_{\eta(\omega)}^{T(\omega)} Q(t)dt \\ &=& \eta_{adj}(\omega)+ f(\eta(\omega)) \end{array} $$

where *f* is a function of *η*(*ω*). Thus, *T*_*adj*_(*ω*) is dependent of *η*(*ω*). □

### **Proposition 2**

The random variables $\phantom {\dot {i}\!}T_{{adj}_{ji}}$ and $\phantom {\dot {i}\!}\eta _{{adj}_{ji}}$ are independent.

### *Proof*

Let

$$\begin{array}{@{}rcl@{}} T_{adj}(\omega) &=& \int_{0}^{T(\omega)} Q(t)dt \\ &=& H[\!T(\omega)] \end{array} $$

and

$$\begin{array}{@{}rcl@{}} \eta_{adj}(\omega) &=& \int_{0}^{\eta(\omega)} Q(t)dt \\ &=& H[\!\eta(\omega)] \end{array} $$

where *H* is an invertible Borel function (hence monotone). Since that *T*(*ω*) and *η*(*ω*) are independent, consequently *H*[ *T*(*ω*)] and *H*[ *η*(*ω*)] are independent. Thus, $\phantom {\dot {i}\!}T_{{adj}_{ji}}$ and $\phantom {\dot {i}\!}\eta _{{adj}_{ji}}$ are independent. □

### **Proposition 3**

The random variables *C*_*ji*_ and *η*_*ji*_ are dependent.

### *Proof*

To simplify the notation, let assume the following be for an individual *i* on a therapeutic arm *j*. One has:

$$ C(\omega) = \left\{\begin{array}{ll} \int_{0}^{T(\omega)} C_{k}(t) dt & \text{if} \,\, T(\omega)\leq \eta(\omega); \\ \int_{0}^{\eta(\omega)} C_{k}(t)dt & \text{if} \,\, \eta(\omega) \leq T(\omega). \end{array}\right. $$

Then, with *E*(*ω*)= inf(*η*(*ω*),*T*(*ω*)), 

Thus, assuming that *T*(*ω*) is independent from *η*(*ω*), one sees that *η*(*ω*) and *C*(*ω*) are dependent. □

## Abbreviations

CEA: Cost-effectiveness analysis
DGP: Data generating process
DIC: Deviance information criterion
ICER: Incremental cost-effectiveness ratio
INB: Incremental net benefit
IPCW: Inverse probability censoring weighted
QALY: Quality adjusted life years
